# Model responses to CO_2_ and warming are underestimated without explicit representation of Arctic small‐mammal grazing

**DOI:** 10.1002/eap.2478

**Published:** 2021-12-08

**Authors:** Edward B. Rastetter, Kevin L. Griffin, Rebecca J. Rowe, Laura Gough, Jennie R. McLaren, Natalie T. Boelman

**Affiliations:** ^1^ The Ecosystems Center Marine Biological Laboratory Woods Hole Massachusetts 02543 USA; ^2^ Department of Ecology, Evolution and Environmental Biology Columbia University New York New York 10027 USA; ^3^ Department of Earth and Environmental Sciences Columbia University Palisades New York 10964 USA; ^4^ Lamont‐Doherty Earth Observatory Columbia University Palisades New York 10964 USA; ^5^ Natural Resources and the Environment University of New Hampshire Durham New Hampshire 03824 USA; ^6^ Department of Biological Sciences Towson University Towson Maryland 21252 USA; ^7^ Department of Biological Sciences University of Texas at El Paso El Paso Texas 79968 USA

**Keywords:** Arctic tundra, biogeochemistry, carbon cycling, carbon–nitrogen ecosystem model, climate change, nitrogen cycling, population cycles, small‐mammal herbivores

## Abstract

We use a simple model of coupled carbon and nitrogen cycles in terrestrial ecosystems to examine how “explicitly representing grazers” vs. “having grazer effects implicitly aggregated in with other biogeochemical processes in the model” alters predicted responses to elevated carbon dioxide and warming. The aggregated approach can affect model predictions because grazer‐mediated processes can respond differently to changes in climate compared with the processes with which they are typically aggregated. We use small‐mammal grazers in a tundra as an example and find that the typical three‐to‐four‐year cycling frequency is too fast for the effects of cycle peaks and troughs to be fully manifested in the ecosystem biogeochemistry. We conclude that implicitly aggregating the effects of small‐mammal grazers with other processes results in an underestimation of ecosystem response to climate change, relative to estimations in which the grazer effects are explicitly represented. The magnitude of this underestimation increases with grazer density. We therefore recommend that grazing effects be incorporated explicitly when applying models of ecosystem response to global change.

## Introduction

Despite evidence that animals can influence ecosystem carbon (C) and nutrient cycles (Schmitz 2014), the explicit incorporation of animals into terrestrial biogeochemical models is rare (Metclafe and Olofsson 2015). To maintain mass balance in these models without explicit representation of animals, the effects of animals have to be implicitly aggregated into other biochemical processes through model calibration (e.g., animal respiration included with other heterotrophic respiration). However, animal‐mediated processes can behave differently from the processes with which they are aggregated. For example, combining microbial and mammal respiration into a single value for heterotrophic respiration can cause problems, because warming generally increases respiration in microbes, but can slow respiration in mammals if the warming reduces the energy needed to maintain body temperature (Batzli et al. 1980). Here we examine the effects of aggregating grazer‐mediated processes in with other biogeochemical processes when modeling ecosystem response to elevated carbon dioxide (CO_2_) and warming. We use small‐mammal grazers in Arctic tundra as an example. However, the principles are general, and we conclude with a discussion of how our results might apply more generally to other grazers and other ecosystems.

Recent studies suggest that animals influence the response of tundra to climate change (Tuomi et al. 2019, Petit Bon et al. 2020). Experimental manipulations conducted across a range of tundra ecosystems have shown that, while warming or fertilization typically enhances above ground productivity and nutrient cycling in tundra, the presence of herbivores – including rodents, geese, and ungulates – can dampen or negate this response (e.g., Grellman 2002, Post and Pedersen 2008, Sjögersten and van der Wal 2008, Rinnan and Stark 2009, Cahoon et al. 2011, Kaarlejärvi and Hoset 2015, Leffler et al. 2019) or might enhance productivity (Gough et al. 2012). Furthermore, observational studies have shown that trophic interactions on the tundra strengthen under warmer conditions (McKinnon et al. 2010, Legagneux 2012), suggesting that animal influences on C cycling might be stronger in the future.

In other terrestrial ecosystems, animals are known to affect C and nutrient cycling (McNaughton 1985, McLaren and Jefferies 2004, Wilkinson and Sherratt 2016). The direct effects of animals vary among ecosystems, type of herbivore, and plant growth form (Jai et al. 2018) but have historically been thought of as small, relative to plant and microbial processes (e.g., Hairston and Smith 1960). Nevertheless, animals can accelerate nutrient cycles and influence plants and microbes indirectly by mediating chemical and biological processes and altering community structure and can thereby have a large influence on ecosystem C and nutrient processing (Pastor et al. 1988, Wardle et al. 2004, Zimov et al. 2009, Metcalfe 2014, Schmitz et al. 2014).

Although herbivore–vegetation models have been made for other ecosystems (e.g., Seagle and McNaughton 1993, Bennett 2003), we are aware of only one vegetation‐dynamics model – ArcVeg – that explicitly addresses the effect of an Arctic herbivore (caribou) on tundra biogeochemistry (Yu et al. 2011). This model indicates that grazing dampens the increase in plant biomass expected from warming soils and the consequent increase in nutrient cycling (Yu et al. 2011). These results suggest that the explicit inclusion of grazers in biogeochemical models could be necessary for predicting tundra responses to climate change.

Other modeling studies have addressed Arctic biogeochemical responses to climate change, but without the explicit representation of the effects of animals as separate from other C and nutrient‐cycling processes. These biogeochemical models indicate significant long‐term impacts of elevated CO_2_ and warming, but the model predictions differ on how these responses will ultimately affect net C source vs. sink activity. This source–sink disparity is due to uncertainty in the balance between elevated autotrophic and heterotrophic respiration (source) resulting from warming vs. enhanced photosynthesis (sink) resulting from the direct effects of elevated CO_2_ and warming on production and from the acceleration of nutrient cycles by warming (McKane et al. 1997, Rastetter and Ågren 1997, McGuire 2012, Pearce et al. 2015, Jiang et al. 2017). This trade‐off between source vs. sink activity is likely to be confounded by Arctic herbivores.

From the perspective of ecosystem biogeochemistry, aggregating herbivore effects in with other processes can be justified, because grazers perform several nutrient‐cycling processes that parallel other plant and microbial processes within ecosystems. Here we examine the aggregation effects for four such processes in relation to the response of ecosystems to elevated CO_2_ and warming:

1. Grazing mediates the transfer of plant C to detritus and soil organic matter (soil), and thereby acts in parallel with tissue senescence and litter fall.
2. Similarly, grazing transfers plant N to soil organic matter in parallel with tissue senescence and litter fall, but does so before the plants can resorb N.
3. Consumption of plant material and subsequent heterotrophic respiration by grazers parallels litter fall and the subsequent heterotrophic respiration resulting from processing of soil organic matter by microbes and other detritivores.
4. Finally, metabolic processing of plant matter consumed by grazers produces dissolved labile N in urine in parallel with litter fall and microbially mediated mineralization.

Even though these processes act in parallel, the grazer‐mediated and non‐grazer‐mediated processes might respond differently to climate change, or even in opposite directions. Furthermore, the cyclic dynamics of small‐mammal grazers in the Arctic might complicate the relative contributions of these parallel processes to ecosystem responses to elevated CO_2_ and climate change. Based on the modeling analysis we present below:

1. We hypothesize that aggregating the effects of small‐mammal grazers with other C and N cycling processes results in an underestimation of tundra responses to elevated CO_2_ and warming. For our model, after 100 years the underestimation of C sequestration in tundra ecosystems in response to elevated CO_2_ and warming is 50–80% relative to estimations in which the grazer effects are explicitly represented.
2. We hypothesize that although three‐to‐four‐year cycles in the density of small‐mammal grazers have measurable short‐term effects of tundra biogeochemistry (e.g., Olofsson and Tømmervik 2012), densities averaged over the grazer cycles can be used to assess long‐term responses of tundra to elevated CO_2_ and warming.

We use a simple model of coupled C and N cycles in ecosystems applied to the effects of small‐mammal grazers on the responses of moist acidic tundra to elevated CO_2_ and warming. Most of the data we use are for lemmings and voles, but the model applies to generic small‐mammal grazers in the Arctic, which we refer to as “voles” for simplicity. We apply the model both with vole densities explicitly represented and with vole densities unspecified, but their effects implicitly subsumed into other biogeochemical processes. In all applications of the model, we assume voles are present on the landscape. The model applications differ only in the way that vole and other biogeochemical processes are separated from one another.

## Methods

### Model

We use a model developed by Rastetter et al. (2020) to examine recovery of ecosystems from disturbances that remove vegetation (Box 1; Rastetter et al. 2021*a*). We have modified that model to account for temperature sensitivity of six metabolic processes (photosynthesis, autotrophic and heterotrophic respiration, plant and microbial N uptake, and N mineralization). We have also modified it to account for the effects of voles on the transfer of C and N from vegetation to soil organic matter and the transfer of N in urine from vegetation to inorganic N (although not all N in urine is inorganic, it is labile, and we treat it as inorganic). The basic model is fully described in Rastetter et al. (2020); here we describe only the changes to that model for the current analyses.

**Model equations**

1 Variables and parameters defined in Table 1.

**Table:** 

**Mass‐balance equations**			
1	$\frac{dB_{C}}{\mathrm{dt}}=P_{s}-L_{\text{itC}}-R_{a}-G_{C}$	2	$\frac{dB_{N}}{\mathrm{dt}}=U_{N}-L_{\text{itN}}-G_{N}$
3	$\frac{dD_{C}}{\mathrm{dt}}=L_{\text{itC}}+L_{\text{VC}}-R_{h}-Q_{\text{CR}}$	4	$\begin{array}{c} \frac{dD_{N}}{\mathrm{dt}}=L_{\text{itN}}+U_{\text{Nm}}+L_{\text{VN}}\\ -N_{\min}-Q_{\text{NR}} \end{array}$
		5	$\begin{array}{c} \frac{\mathrm{dN}}{\mathrm{dt}}=N_{\text{in}}+N_{\min}+V_{\text{UN}}\\ -U_{N}-U_{\text{Nm}}-Q_{\text{DIN}} \end{array}$
**Allometry and stoichiometry constraints**			
6	$S=B_{C}\frac{\left(\propto B_{C}+1\right)}{\left(\gamma B_{C}+1\right)}$	7	$\Psi =\frac{B_{C}}{B_{N}q_{B}}$
8	$\Phi =\frac{D_{C}}{D_{N}q_{D}}$		
**Process equations**			
**Carbon**		**Nitrogen**	
9	$P_{s}=\frac{g_{C}SC_{a}}{\Psi \left(k_{C}+C_{a}\right)}Q_{10\text{Ps}}^{T/10}$	10	$U_{N}=\frac{g_{N}\Psi SN}{k_{N}+N}Q_{10U}^{T/10}$
11	$L_{\text{itC}}=m_{\text{CB}}B_{C}$	12	$L_{\text{itN}}=\frac{m_{\text{NB}}}{\Psi }B_{N}$
13	$R_{a}=r_{B}B_{C}\Psi Q_{10\text{Ra}}^{T/10}$	14	$U_{\text{Nm}}=\frac{g_{\text{Nm}}\Phi D_{C}N}{k_{\text{Nm}}+N}Q_{10\text{Um}}^{T/10}$
15	$G_{C}=\left(n_{V}+g_{V}-\varepsilon_{V}\left(T-T_{0}\right)\right)V/10,000$	16	$G_{N}=G_{C}/q_{V}$
17	$R_{V}=r_{V}\left(G_{C}-n_{V}V/10,000\right)$	18	$V_{\text{UN}}=m_{\text{NV}}V/10,\;000$
19	$L_{\text{VC}}=G_{C}-R_{V}$	20	$L_{\text{VN}}=G_{N}-V_{\text{UN}}$
21	$R_{h}=r_{D}D_{C}\Phi Q_{10\text{Rh}}^{T/10}$	22	$N_{\min}=\frac{m_{\text{Nm}}D_{N}}{\Phi }Q_{10m}^{T/10}$
23	$Q_{\text{CR}}=q_{\text{DOM}}Q_{\text{NR}}$	24	$Q_{\text{NR}}=\beta_{\text{NR}}D_{N}$
		25	$Q_{\text{DIN}}=\beta_{N}N$

**Table 1:** Model variables and parameters.

	Symbol	Value	Units
C and N stocks			
Vegetation C	*B*_C_	878	g C/m^2^
Detritus and soil organic C	*D*_C_	19,452	g C/m^2^
Vegetation N	*B*_N_	20.6	g N/m^2^
Detritus and soil organic N	*D*_N_	831	g N/m^2^
Inorganic N	*N*	0.27	g N/m^2^
Processes and constraints			
Allometric constraint	*S*	243.75	g C/m^2^
Vegetation stoichiometric constraint	Ψ	1	None
Soil stoichiometric constraint	Φ	1	None
Photosynthesis	*P*_s_	430	g C·m^−2^·yr^−1^
Autotrophic respiration	*R*_a_	215	g C·m^−2^·yr^−1^
Litter‐fall C	*L*_itC_	215	g C·m^−2^·yr^−1^
Heterotrophic respiration (excluding voles)	*R*_h_	213.07	g C·m^−2^·yr^−1^
Vegetation N uptake	*U*_N_	5.3800	g N·m^−2^·yr^−1^
Litter‐fall N	*L*_itN_	5.3800	g N·m^−2^·yr^−1^
Gross N mineralization	*N*_min_	19.7310	g N·m^−2^·yr^−1^
N immobilization	*U*_Nm_	14.4824	g N·m^−2^·yr^−1^
Inorganic N losses	*Q*_DIN_	0.0016	g N·m^−2^·yr^−1^
Refractory N losses	*Q*_NR_	0.1314	g N·m^−2^·yr^−1^
Refractory C losses	*Q*_CR_	1.93	g C·m^−2^·yr^−1^
C removed from vegetation by voles	*G*_C_	0	g C·m^−2^·yr^−1^
Vole respiration	*R*_V_	0	g C·m^−2^·yr^−1^
C added to soil organic matter by voles	*L*_VC_	0	g C·m^−2^·yr^−1^
N removed from vegetation by voles	*G*_N_	0	g N·m^−2^·yr^−1^
C added to soil organic matter by voles	*L*_VN_	0	g N·m^−2^·yr^−1^
Vole N transfer vegetation to inorganic soil	*V*_UN_	0	g N·m^−2^·yr^−1^
Driver variables			
Atmospheric CO_2_	*C*_a_	400	μmol/mol
Temperature	*T*	10	°C
N inputs	*N*_in_	0.1330	g N·m^−2^·yr^−1^
Voles	*V*	0	voles/ha
Parameters			
Allometric parameter 1	α	0.002231	m^2^·g^−1^ C
Allometric parameter 2	γ	0.01100	m^2^·g^−1^ C
Optimum vegetation C:N	*q*_B_	42.62	g C·g^−1^ N
Optimum soil C:N	*q*_D_	23.41	g C·g^−1^ N
Photosynthesis rate parameter	*g*_C_	1.423	yr^−1^
CO_2_ half‐saturation constant	*k*_C_	100.0	μmol/mol
Photosynthesis Q‐10	*Q*_10Ps_	1.550	None
Autotrophic respiration constant	*r*_B_	0.09069	yr^−1^
Autotrophic respiration Q‐10	*Q*_10Ra_	2.700	None
Vegetation C turnover rate constant	*m*_CB_	0.2449	yr^−1^
Vegetation N‐uptake rate parameter	*g*_N_	0.05191	g N·g^−1^ C·yr^−1^
Vegetation N half‐saturation constant	*k*_N_	1.000	g N/m^2^
Vegetation N‐uptake Q‐10	*Q*_10U_	2.000	None
Vegetation N turnover rate constant	*m*_NB_	0.2612	yr^−1^
Heterotrophic respiration constant	*r*_D_	0.003651	yr^−1^
Heterotrophic respiration Q‐10	*Q*_10Rh_	3.000	None
Microbial N‐uptake rate parameter	*g*_Nm_	0.001796	g N·g^−1^ C·yr^−1^
Microbial N half‐saturation constant	*k*_Nm_	1.000	g N/m^2^
Microbial N‐uptake Q‐10	*Q*_10Um_	1.950	None
Vole nesting material	*n*_V_	22	g C·vole^−1^·yr^−1^
Vole C ingestion rate	*g*_V_	3,512	g C·vole^−1^·yr^−1^
Temperature slope vole metabolism	ε_V_	52	g C·vole^−1^·°C^−1^·yr^−1^
Summer to annual temperature correction	*T*_0_	10	°C
C:N of vole forage and nest material	*q*_V_	19.15	g C·g^−1^ N
Vole base respiration rate	*r*_V_	0.3	None
Vole urine N production rate	*m*_NV_	11.00	g N·vole^−1^·yr^–1^
Soil organic N turnover constant	*m*_Nm_	0.01099	yr^–1^
Soil organic N turnover Q‐10	*Q*_10m_	2.160	None
C:N of DOM loss	*q*_DOM_	14.69	g C·g^−1^ N
N loss‐rate parameter	β_N_	0.005926	yr^−1^
Refractory N loss parameter	β_NR_	0.0001581	yr^−1^

Notes

Variable values are for the initial steady state with the aggregated representation of vole effects. Ψ and Φ are assumed to equal 1 under this steady state. *Q*_10_ values are as reported in the main text. Other values are from Pearce et al. (2015) or are fitted to analogous functions in Pearce et al. (2015). Parameters are listed to four significant digits. DOM, dissolved organic matter.

#### Temperature response of metabolic processes

Because we use an annual time step in the model (i.e., no seasonality) and restrict warming to 5°C above current temperatures, we use a simple *Q*_10_ function to simulate temperature responses rather than more complex formulations (e.g., Heskel 2016 or Carey 2016). We have therefore modified the photosynthesis, autotrophic respiration, heterotrophic respiration, plant N uptake, microbial N uptake, and N mineralization in the Rastetter et al. (2020) model to increase exponentially with warming (Box 1: Eqs. 9, 10, 13, 14, 21, 22).

#### Vole grazing

**Fig. 1:**
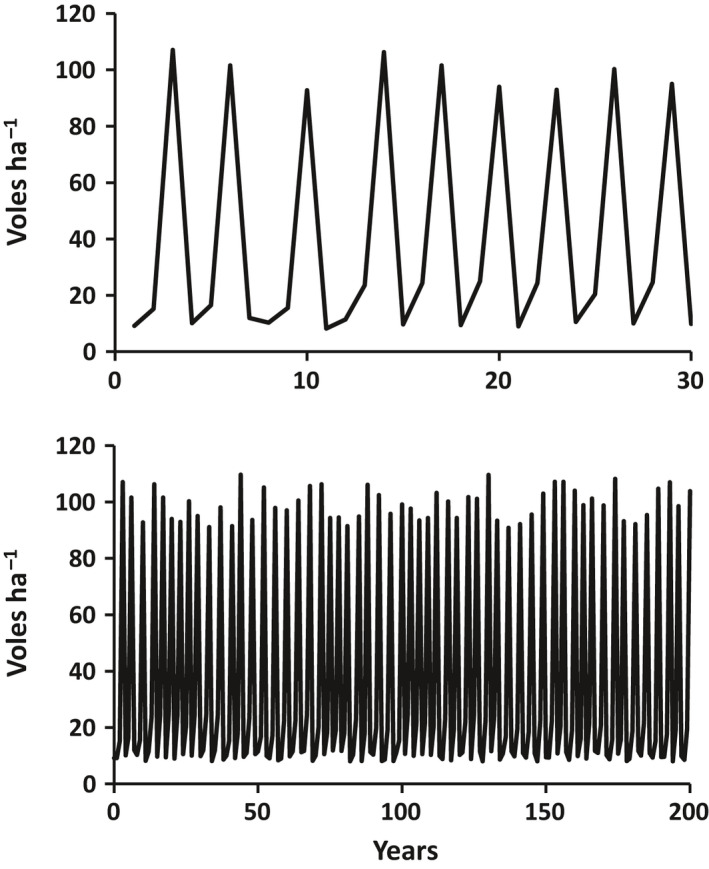
Vole cycle used to simulate ecosystem response to grazing. Vole abundance is randomly generated with peaks every three or four years, with abundances at the peak ranging from 90 to 110 voles/ha, minimum abundances ranging from 8 to 12 voles/ha, and a mean vole abundance of 40 voles/ha. Upper panel shows the first 30 years of the time series. Bottom panel is the full 200‐year time series.

We drive the model by specifying vole density in each year (*V*). Consistent with values and cycle frequency reported in the literature (Batzli et al. 1980, Krebs and Boonstra 1995, Korpimaki et al. 2004, Pitelka and Batzli 2007, Krebs 2013, Ehrich 2020), we use a randomly generated time series of vole abundance with peaks every three or four years, with abundances at the peak ranging from 90 to 110 voles/ha, minimum abundances ranging from 8 to 12 voles/ha, and a mean vole abundance over the full time series of 40 voles/ha (Fig. 1). We chose to take this prescribed approach to vole density because the drivers of vole cycles are not fully understood (Korpimaki et al. 2004, Prevedello et al. 2013, Oli 2019) and are likely to include a top‐down component (Pitelka and Tomich 1955, Hairston et al. 1960, Krebs 2013), which is well beyond the domain of our model. For convenience, we specify vole density in voles/ha and correct to m^−2^ units by dividing by 10,000 m^2^/ha (Eqs. 15, 17, 18).

The removal of C from vegetation for nest building and ingestion are lumped into a single process for our model (Eq. 15). We assume this C removal is proportional to the specified vole density but decrease the per capita rate of ingestion with warming to account for decreased energy requirements to maintain body temperature (Eq. 15; Batzli et al. 1980). Vole respiration is proportional to the ingestion component of C removal from vegetation by voles and therefore also declines with warming (Eq. 17). We do not account for other temperature responses such as those associated with cold or heat stress. We assume a constant C:N ratio of material removed from vegetation by grazers (Eq. 16) but, because of the respiration loss of C and the N transferred to inorganic N in urine, the C:N of material removed from vegetation and the C:N of material added to the soil organic matter differ. Finally, we assume urine losses of N are proportional to vole density (Eq. 18).

### Model calibration

We calibrate the model to be consistent with the C and N stocks and process rates compiled by Pearce et al. (2015) for the Multiple Element Limitation (MEL) model (Table 1). Because voles are part of the ecosystem, the effects of voles on tundra C and N stocks and fluxes are implicitly included in the data compiled by Pearce et al. (2015). Therefore, by using these data to calibrate (fit) the model without explicit vole representation, we are implicitly aggregating those vole effects in with the parallel ecosystem processes described above. In the calibrations in which voles are explicitly represented, we assume a constant vole density and that the ecosystem is in steady state. We then specify the vole effects directly and subtract these effects from the parallel ecosystem processes before calibration (Table 2). The combined rates of vole‐mediated processes plus the parallel ecosystem processes are therefore identical in all calibrations (rows labeled “Total” in Table 2), therefore providing the basis of comparison for assessing the consequences of explicit vs. aggregated representation of vole grazing.

**Table 2:** Variable and parameter changes to accommodate the effect of explicit representation of voles.

Symbol	Total or PAR	Vole effects aggregated in with other processes	Explicit vole representation with 40 voles/ha	Explicit vole representation with 100 voles/ha	Units
*L*_itC_		215	200.864 (−6.6%)	179.66 (−16.4%)	g C·m^−2^·yr^−1^
*G*_C_		0	14.136	35.34	g C·m^−2^·yr^−1^
	Total	215	215	215	g C·m^−2^·yr^−1^
*m*_CB_	PAR	0.2449	0.2288 (−6.6%)	0.2046 (−16.4%)	yr^−1^
*R*_h_		213.07	208.8556 (−2.0%)	202.534 (−4.9%)	g C·m^−2^·yr^−1^
*R*_v_		0	4.2144	10.536	g C·m^−2^·yr^−1^
	Total	213.07	213.07	213.07	g C·m^−2^·yr^−1^
*r*_D_	PAR	0.003651	0.003658 (−2.0%)	0.003471 (−4.9%)	yr^−1^
*L*_itN_		5.38	4.6418 (−13.7%)	3.5346 (−34.3%)	g N·m^−2^·yr^−1^
*G*_N_		0	0.7382	1.8454	g N·m^−2^·yr^−1^
	Total	5.38	5.38	5.38	g N·m^−2^·yr^−1^
*m*_NB_	PAR	0.2612	0.2253 (−13.7%)	0.1716 (−34.3%)	yr^−1^
*V*_UN_		0	0.044	0.110	g N·m^−2^·yr^−1^
*N*_min_		19.731	19.687 (−0.2%)	19.621 (−0.6%)	g N·m^−2^·yr^−1^
	Total	19.731	19.731	19.731	g N·m^−2^·yr^−1^
*m*_Nm_	PAR	0.01099	0.01097 (−0.2%)	0.01093 (−0.6%)	yr^−1^

Notes

Values in parentheses are the percent change from the values used in the implicit‐vole representation with vole effects aggregated in with parallel ecosystem processes. “Total” is the total of the vole‐mediated and the parallel ecosystem process in the two preceding rows. “PAR” is the parameter in the equation for the parallel process in the preceding rows that was modified to accommodate explicit representation of voles.

In the calibrations, first we set allometric, C:N, *Q*_10_, half‐saturation, and vole‐related parameters (derivation of these parameter values is presented in Appendix S1). We then set the rate parameters for each process so that flux rates are consistent with rates reported in Pearce et al. (2015). Because annual rates of plant and microbial processes are dominated by growing‐season rates, we use average summer temperature (10°C) to calibrate the model; in any case, because of the *Q*_10_ formulation, once calibrated to a specified temperature, model responses are sensitive to changes in temperature, not to the temperature value itself. For vole processes, we correct this summer temperature to average annual temperature with an off‐set (Eq. 15).

We made three calibrations (Tables 3, 4). In calibration I, vole densities are not explicitly specified, and we assume that vole‐mediated processes can be implicitly represented by aggregating them with the parallel biogeochemical processes described in the introduction above (Table 2). In this calibration, we therefore set the number of voles (*V*) in the model to zero but incorporate the effects of voles in with the parallel processes through the calibration. In calibration II, we set the number of voles to 40 voles/ha so that vole‐mediated processes are explicitly represented, and the number of voles is the mean abundance of voles we use in our simulated vole cycle (described above). In calibration III, we set the number of voles to 100 voles/ha, which is the mean peak vole abundance in our simulated vole cycle. In calibration III, the parallel ecosystem process rates are decreased proportionally more than in calibration II to account for the higher vole density (Table 2).

**Table 3:** Calibrations.

Calibration	Vole representation	Vole density
I	Vole effects aggregated in with other processes	Unspecified vole density, but vole effects subsumed into litter‐fall C and N, heterotrophic respiration, and N mineralization in the calibration (Table 2)
II	Explicit vole representation	40 voles/ha
III	Explicit vole representation	100 voles/ha

**Table 4:** Simulations.

Simulation	Calibration	Description	Figure
Set 1			
1	II	Constant 40 voles/ha	Fig. 2 dotted black lines
2	II	Voles cycling as in Fig. 1	Fig. 2 cycling black solid lines
3	II	Voles cycling as in Fig. 1 for 10 yr then held constant at 100 voles/ha	Fig. 2 dashed red lines
4	II	Voles cycling as in Fig. 1 for 10 yr then held constant at 0 voles/ha	Fig. 2 dash‐dotted blue lines
Set 2			
5	I	Vole density unspecified, linear increase of CO_2_ from 400 to 800 μmol/mol over 100 yr	Figs. 4, 5, 6 dotted line, left column
6	I	Vole density unspecified, linear increase in temperature from 10 to 15°C over 100 yr	Figs. 4, 5, 6 dotted line, middle column
7	I	Vole density unspecified, linear increase of CO_2_ from 400 to 800 μmol/mol and temperature from 10 to 15°C over 100 yr	Figs. 4, 5, 6 dotted line, right column
8	II	Vole density cycling as in Fig. 1, linear increase of CO_2_ from 400 to 800 μmol/mol over 100 yr	Figs. 4, 5, 6 blue solid line, left column
9	II	Vole density cycling as in Fig. 1, linear increase in temperature from 10 to 15°C over 100 yr	Figs. 4, 5, 6 blue solid line, middle column
10	II	Vole density cycling as in Fig. 1, linear increase of CO_2_ from 400 to 800 μmol/mol and temperature from 10 to 15°C over 100 yr	Figs. 4, 5, 6 blue solid line, right column
11	III	Constant 100 voles/ha, linear increase of CO_2_ from 400 to 800 μmol/mol over 100 yr	Figs. 4, 5, 6 dashed red line, left column
12	III	Constant 100 voles/ha, linear increase in temperature from 10 to 15°C over 100 yr	Figs. 4, 5, 6 dashed red line, middle column
13	III	Constant 100 voles/ha, linear increase of CO_2_ from 400 to 800 μmol/mol and temperature from 10 to 15°C over 100 yr	Figs. 4, 5, 6 dashed red line, right column

All but four of the parameters have the same values in all three calibrations. To maintain the same steady state in all three calibrations, we adjust the values of these four parameters to compensate for how voles are represented in the model (*m*_CB_, *m*_NB_, *r*_D_, and *m*_Nm_; rows labeled “PAR” in Table 2). These parameters are adjusted so that the rates of C and N litter losses, heterotrophic soil respiration, and gross N mineralization all decrease to compensate for the parallel vole‐mediated C and N fluxes in calibrations II and III in which voles are explicitly represented. Because we calibrate to the same data set (Pearce et al. 2015), the overall C and N stocks and cycling rates are identical for these three calibrations (rows labeled “Total” in Table 2).

### Simulations

We run a total of 13 simulations in two sets (Tables 3, 4; Rastetter et al. 2021*b*, *c*).

In the first set of simulations, we assume that the average vole density is 40 voles/ha and use calibration II with vole effects explicitly represented (Table 3). We then run four 200‐year simulations with no change in either CO_2_ or temperature. We drive the model with: (1) voles held constant at 40 voles/ha; (2) voles cycling on the three‐to‐four‐year cycle between 8 and 110 voles/ha for 200 years; (3) voles cycling for 10 years, followed by maintenance of a constant vole density of 100 voles/ha (equivalent to adding voles to the ecosystem); and (4) voles cycling for 10 years, followed by maintenance of a constant vole density of 0 voles/ha (equivalent to removing voles from the ecosystem).

This first set of simulations serves two purposes. First, it illustrates the effects of long‐term changes in vole density and thereby draws the distinction between adding or removing voles from calibrating the model assuming high or low vole density. Second, it allows us to assess the potential long‐term effects of voles if their numbers were maintained at high or low levels. We can thereby address the question: “Do the simulated changes in the ecosystem approach their potential changes during peaks and troughs in the vole cycle?”

The second set of simulations is to address our central question about aggregated vs. explicit representations of grazer effects on ecosystem responses to climate change (Table 4). We run nine 100‐year simulations in a two‐factor design. The first factor relates to how vole effects are represented in the three calibrations (Table 3) and vole abundance:

1. Calibration I (aggregated) and vole abundance subsumed in the calibration of the parallel processes and therefore assumed constant but unspecified (although *V* is set to 0 in the model, vole effects are aggregated in with the parallel ecosystem processes).
2. Calibration II (40 voles/ha) and vole abundance cycling on the three‐to‐four‐year cycle between 8 and 110 voles/ha.
3. Calibration III (100 voles/ha) and vole abundance held constant at 100 voles/ha.

The second factor relates to climate change:

1. A linear increase in atmospheric CO_2_ from 400 to 800 μmol/mol over 100 years.
2. A linear increase in temperature from 10 to 15°C over 100 years.
3. A linear increase in both atmospheric CO_2_ from 400 to 800 μmol/mol and temperature from 10 to 15°C over 100 years.

## Results

### Set 1: Effects of vole cycling and adding or removing voles

#### Simulation 1: Effects of holding voles constant at the calibration abundance

**Fig. 2:**
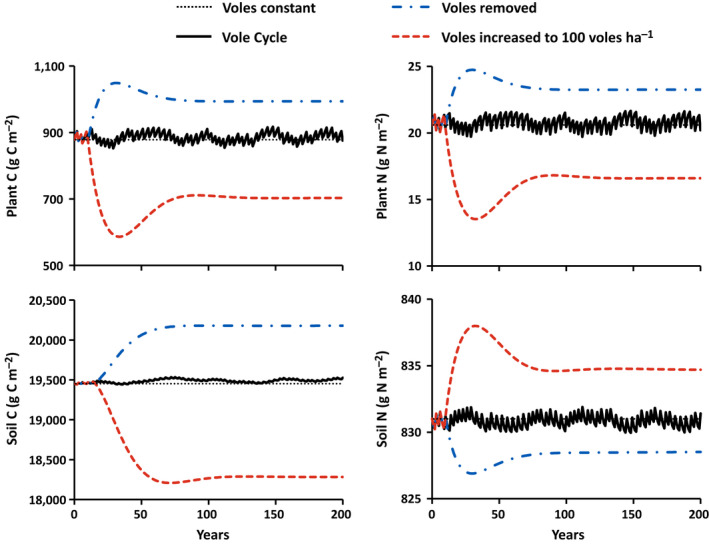
Simulated changes in plant and soil organic carbon (C) and nitrogen (N) in response to constant vole abundance, vole cycling, vole density maintained at 100 voles/ha, and vole removal (see Table 4). The thin dotted black lines are the steady‐state values if vole density is held at 40 voles/ha (simulation 1). Solid black lines are the responses to the vole cycle depicted in Fig. 1 (simulation 2). Dashed red lines are the responses to the same vole cycle and then vole density maintained at 100 voles/ha after year 10 (simulation 3). Dashed‐dotted blue lines are the responses to the same vole cycle and then removal of voles after year 10 (simulation 4).

Because the model was calibrated to a steady state with 40 voles/ha, all ecosystem C and N stocks and fluxes remained constant when vole abundance was held at 40 voles/ha in the 200‐year simulations (dotted horizontal lines in Fig. 2). This simulation only serves to illustrate the stability of the model and to serve as a control to which the other simulations can be compared.

#### Simulation 2: Effects of vole cycling on plant and soil C and N

In the 200‐year simulations with vole abundance cycling, the plant and soil C and N stocks cycle at the same three‐to‐four‐year frequency as the voles (Fig. 2). In addition, there are some longer term dynamics in these stocks associated with the autocorrelated nature of plant production and the legacy of the random variations in the vole cycle. Despite these dynamics, vole cycling does not cause the plant and soil C and N stocks to diverge far from the values to which they are calibrated (dotted and solid lines in Fig. 2).

**Fig. 3:**
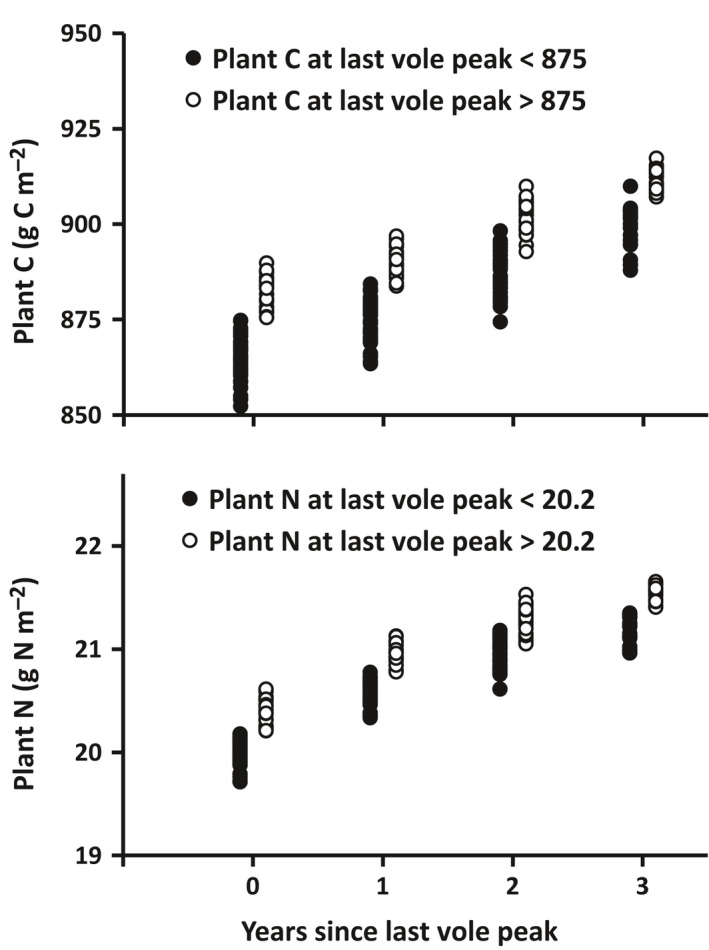
Plant carbon (C) and nitrogen (N) recovery following peak vole abundance in simulation 2. The plant C and N of 875 g C/m^2^ and 20.2 g N/m^2^ were selected to partition the recovery time series into two approximately equal‐sized groups based on their values at the time of the previous vole peak (time 0 on *x* axis). The levels of C and N during this recovery depend not only on peak vole abundance, but also on the degree of recovery following the previous vole cycle. Because the biomass consumed is proportional to vole abundance and not to plant biomass, if plants recover to a higher level following the previous cycle (white dots), then they begin and maintain recovery in the current cycle at a higher level relative to plants that recovered to a lower level in the previous cycle (black dots). This autocorrelation results in the longer term dynamics in Fig. 2 for simulation 2 in which vole abundance cycled. The recovery in any cycle also depends on vole abundance and the duration of the vole cycle (higher plant recovery in a four‐year cycle than a three‐year cycle).

The plant biomass cycles out of phase with the vole cycle. The lowest plant C and N values occur in years of peak vole numbers and the highest plant C and N values occur three or four years after peak vole numbers or the year prior to the subsequent vole peak (Fig. 3). This phase shift in the plant relative to vole cycles as well as the magnitude of the plant C cycle (20–30 g C/m^2^ peak to trough) are roughly consistent with the phase and magnitude of the cycles reported by Olofsson et al. (2012). In addition, the dependence of plant production on plant biomass results in a strong autocorrelation in the plant C and N time series, which in turn results in longer term dynamics less clearly tied to the vole cycle (Fig. 2). This autocorrelation is reflected in the strong positive correlation between the plant C and N and their respective values at the time of the previous peak in vole numbers (open dots remain high and closed dots remain low in Fig. 3).

The dynamics of soil C and N stocks are closely tied to the plant dynamics. Because N inputs to the ecosystem are less than 3% of the annual plant requirement (Table 1), plant recovery from vole outbreaks relies almost exclusively on N from soil organic matter. As a consequence, the three‐to‐four‐year soil N cycles are directly out of phase with plant N cycles and the longer term dynamics are also opposite those in plant N (Fig. 2). Soil C also cycles out of phase with plant C, but the relation is not as strong as it is for N. However, because soil C is ultimately derived from plant C, the longer term dynamics in plant C are paralleled in the soil C following approximately a 9‐year lag (Fig. 2).

#### Simulations 3 and 4: Effects of removing or adding voles

When voles are removed from the ecosystem, plant C and N increase by approximately 13%, or increase by 116 g C/m^2^ and 2.7 g N/m^2^. Because of the reliance of plants on soil N, soil N deceases by almost the same absolute amount as the plants gain, 2.5 g N/m^2^. However, the amount of N in the soil is so large that this loss amounts to only an approximately 0.3% loss. The increase in plant biomass results in higher litter inputs to the soil. Consequently, soil C increases by approximately 4% or 727 g C/m^2^. The gain of soil C and loss of soil N widens the soil C:N ratio by approximately 4%, which in turn increases microbial N immobilization into soil organic matter (effect of Φ in Eq. 14).

When voles are increased and then held constant at 100 voles/ha, plant C and N decrease by approximately 20%, or 175 g C/m^2^ and 4 g N/m^2^. Again, because of the tight cycling of N in the ecosystem, soil N increases by almost the same absolute amount as the plants lose, 3.7 g N/m^2^ (0.4%). The loss of plant biomass translates into lower litter inputs to soil and a large absolute decrease in soil C, 1,170 g C/m^2^ (6%). Because of the increase in soil N and decrease in soil C, the soil C:N narrows by approximately 6%, which in turn decreases microbial N immobilization into soil organic matter (effect of Φ in Eq. 14).

In the simulations in which vole density is increased or decreased and then held constant, it takes 10–20 years for the vole effects to reach their largest deviation from the steady state and another 60–90 years for those effects to stabilize. Because of this long response time, the potential effects of voles on tundra biogeochemistry cannot be approached if vole abundance cycles on a three‐to‐four‐year cycle. Indeed, when voles are cycling, the magnitude of these effects relative to the peak effects of the long‐term increase or decrease in vole abundance is only approximately 12% for plant C, 2% for soil C, and 20% for both plant and soil N.

### Set 2: Effects of aggregated vs. explicit representations of vole effects

#### Simulations 5, 8, and 11: Responses to increasing CO_2_

**Fig. 4:**
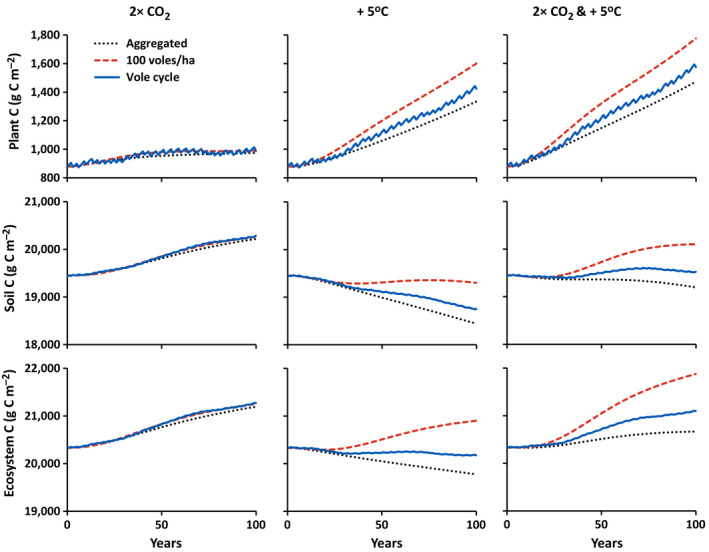
Simulated changes in plant, soil, and total ecosystem C with a linear increase in CO_2_ from 400 to 800 μmol/mol over 100 years, a linear warming from 10 to 15°C over 100 years, and both a linear increase in CO_2_ from 400 to 800 μmol/mol and a linear warming from 10 to 15°C over 100 years (see Table 4). Different trajectories indicate responses with vole effects aggregated with other biogeochemical processes (dotted black lines; simulations 5, 6, and 7), voles cycling between 8 and 110 voles/ha on a three‐to‐four‐year cycle (solid blue lines; simulations 8, 9, and 10), and a constant 100 voles/ha (dashed red lines; simulations 11, 12, and 13).

**Fig. 5:**
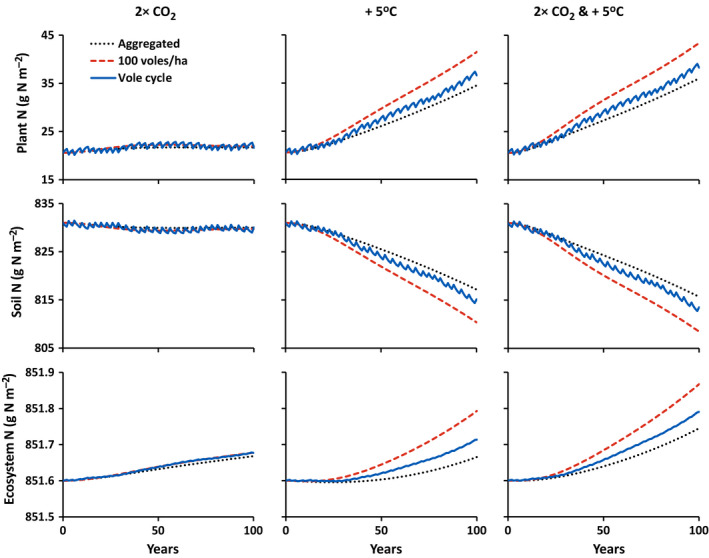
Simulated changes in plant, soil, and total ecosystem N with a linear increase in CO_2_ from 400 to 800 μmol/mol over 100 years, a linear warming from 10 to 15°C over 100 years, and both a linear increase in CO_2_ from 400 to 800 μmol/mol and a linear warming from 10 to 15°C over 100 years (see Table 4). Different trajectories indicate responses with vole effects aggregated with other biogeochemical processes (dotted black lines; simulations 5, 6, and 7), voles cycling between 8 and 110 voles/ha on a three‐to‐four‐year cycle (solid blue lines; simulations 8, 9, and 10), and a constant 100 voles/ha (dashed red lines; simulations 11, 12, and 13).

**Fig. 6:**
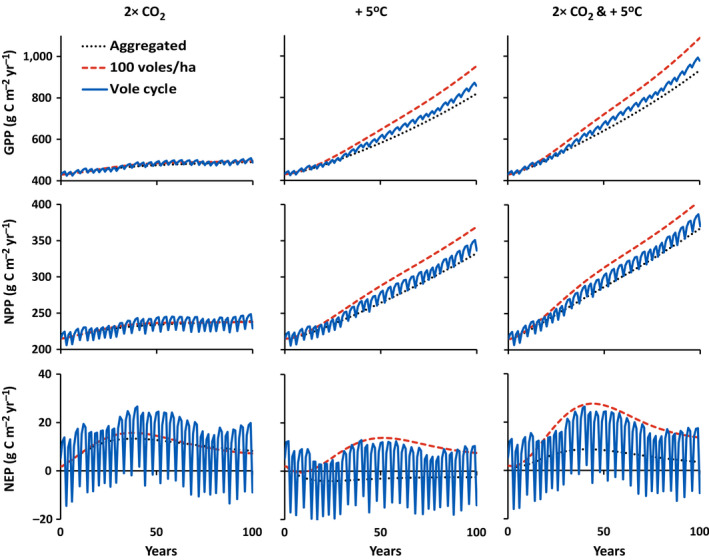
Simulated changes in gross primary (GPP), net primary (NPP), and net ecosystem production (NEP) with a linear increase in CO_2_ from 400 to 800 μmol/mol over 100 years, a linear warming from 10 to 15°C over 100 years, and both a linear increase in CO_2_ from 400 to 800 μmol/mol and a linear warming from 10 to 15°C over 100 years (see Table 4). Different trajectories indicate responses with vole effects aggregated with other biogeochemical processes (dotted black lines; simulations 5, 6, and 7), voles cycling between 8 and 110 voles/ha on a three‐to‐four‐year cycle (solid blue lines; simulations 8, 9, and 10), and a constant 100 voles/ha (dashed red lines; simulations 11, 12, and 13).

Predicted responses to increased CO_2_ do not differ substantially between the aggregated model in which vole effects are implicitly aggregated in with other biogeochemical processes through model calibration (calibration I) and the distributed model in which vole effects are explicitly represented (calibrations II and III: Figs. 4, 5, 6). The only process affected by elevated CO_2_ is photosynthesis. However, because the plants are strongly N limited, elevated CO_2_ increases net primary production (NPP) by only 10–11% in both aggregated and distributed simulations (Fig. 6). This increase in production translates into an approximately 11–12% increase in biomass, again in both aggregated and distributed simulations (Fig. 4). The increase in production results in only about a 4% increase in soil C in all the simulations. This increase in soil C is a small relative change but, because soil has such a large fraction of the organic matter, it is a large absolute change amounting to about 90% of the total change in ecosystem C.

The amount of N entering the ecosystem is too small to support even the small gain in plant C in response to elevated CO_2_. The gain is instead supported by a net transfer of 0.9–1.3 g N/m^2^ from soil to plants over the 100‐year simulations. The amount of N transferred from soil to plants is about the same in all three simulations (Fig. 5). Elevated CO_2_ increases the C:N ratio of the plants, which in turn increases N uptake (effect of Ψ in Eq. 10). However, the increase in soil C:N resulting from increased litter inputs also increases microbial N uptake (effect of Φ in Eq. 14). This competition between plants and microbes for N limits the ecosystem response to elevated CO_2_. Again, the effects of aggregated vs. explicit representation of voles on this response to elevated CO_2_ are negligible (Figs. 4, 5, 6).

#### Simulations 6, 9, and 12: Responses to warming

In contrast, the effects of aggregated vs. explicit representation of voles on the response to warming are large (Figs. 4, 5, 6). Warming not only stimulates photosynthesis, it also stimulates autotrophic and heterotrophic respiration, and, more importantly, it stimulates the N cycle in three places: (1) N mineralization, (2) microbial N uptake, and (3) plant N uptake. A major effect of this stimulation of the N cycle is an increase in net N mineralization, a resulting relaxation of N limitation on plant growth, and a large increase in plant biomass. The increase in plant production increases litter inputs to soils, which in turn mitigates soil C losses. In addition, the increased production allows the ecosystem to accumulate a small amount of N (<0.3 g N/m^2^; Fig. 6). The effects of this chain of events are much stronger in the simulations in which voles are explicitly represented than in the simulations with the aggregated model and the effects are stronger when the model is calibrated assuming higher vole densities (response stronger for calibration III [100 vole/ha] than for calibration II [40 voles/ha]). Therefore, explicit representation of vole effects results in an amplification of the predicted transfer of N from soil to plants, larger predicted gains in plant C or higher predicted retention of soil C, and higher predicted rates of gross primary production (GPP), NPP, and net ecosystem production (NEP). If the simulations are run with vole density held constant at 40 voles/ha, the C and N stocks and fluxes follow the same general patterns as in the simulations with the three‐to‐four‐year vole cycle (data not shown). The size of the differences between the simulations with constant 40 voles/ha and cycling vole density are about the same as those of the cycle simulation from the steady state with no climate change (Fig. 2). In addition, the temperature effects on vole consumption and respiration (Box 1, Eqs. 15, 17) have only a small effect on this general pattern (simulations rerun with ε_V_ = 0 resulted in <2% difference in C and N stocks; data not shown).

#### Simulations 7, 10, and 13: Responses to increasing CO_2_ and warming

The effects of elevated CO_2_ and warming are slightly amplified when the two are combined (the two interact synergistically). Under both elevated CO_2_ and warming, GPP is 12% and NPP is 8% higher than the sum of the changes in GPP and NPP under elevated CO_2_ alone and warming alone (Fig. 6). The net transfer of N from soil to plants is about 3% stronger and the increase in plant C is about 7.5% stronger (Figs. 4, 5). Overall, because the response to CO_2_ alone is so much smaller that the response to warming alone, the response to the two combined is dominated by the warming response. The consequences of aggregated vs. explicit representations of vole effects are therefore the same as in the warming simulations.

In our analysis we assume vole density is top‐down controlled and therefore does not increase with plant production. However, if the average vole density during the cycle increases in proportion to NPP (~80% over 100 year), some of the increased production with elevated CO_2_ and warming is consumed and the increase in plant biomass is about 7.6% lower than when the average vole density remains constant (data not shown). The increase in vole density causes soil C to decrease by 0.9% rather than increase by 0.4%.

## Discussion

Our analysis indicates that failure to explicitly represent small‐mammal grazers (voles) in biogeochemical models can result in an underestimation of the response of Arctic ecosystems to climate warming but has only a small effect on the response to elevated CO_2_ (Figs. 4, 5, 6). Underestimation of the warming response increases with the assumed density of voles used to calibrate the model. Although cycling of vole density has short‐term effects on ecosystem stocks and fluxes, it neither amplifies nor dampens the underestimation in the long‐term response to warming.

### Why is the explicit representation so important?

Before addressing this question, we again emphasize the distinction between explicit representation of voles and adding voles to the ecosystem. Adding voles to the ecosystem accelerates nutrient cycling by increasing the transfer of nutrients from plants to soil. Such an acceleration of nutrient cycling might be expected to increase the responsiveness to elevated CO_2_ and warming. However, in our analysis of the response to elevated CO_2_ and warming we do not add voles, we simply change how the voles are represented in the model. Vole effects are either implicitly aggregated in with other processes or they are explicitly represented. In all three of our calibrations, the total amounts of C and N removed from vegetation, the total heterotrophic respiration, and the total mineralization of N are identical (Table 2). Therefore, the *magnitudes* of vole‐mediated processes plus the parallel ecosystem processes are represented identically in all three calibrations. Furthermore, in the analysis in which we did add voles, any acceleration of nutrient cycles by vole activity is transient; our analysis indicates that the net effect of adding voles is to transfer N from plants, with relatively high N turnover, to soil, with slower N turnover (Fig. 2); although it would be impossible to detect the 0.6% change in soil N predicted by our model. Therefore, the long‐term effect of adding voles is to slow the nutrient cycle. Furthermore, adding and maintaining 100 voles/ha resulted in a loss of over one kilogram of C from the ecosystem (Fig. 2). Therefore, the effect of adding grazers is to *decrease* ecosystem C whereas explicitly representing voles in the model is to *increase* the estimate of C gain with climate change (Fig. 4).

### Why are the differences in responses to elevated CO_2_ so small between aggregated vs. explicit representation of vole effects?

**Fig. 7:**
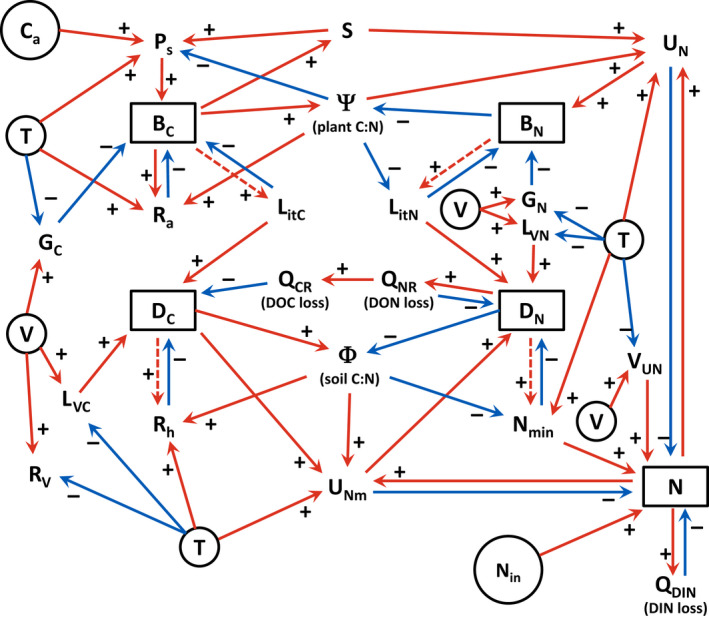
Causal‐chain diagram for the model in Box 1. Arrows indicate causal links: a red arrow marked with a “+” indicates that an increase in the variable at the tail of the arrow will cause an increase in the variable at the head of the arrow; a blue arrow marked with a “−” indicates that and increase in the variable at the tail of the arrow will cause a decrease in the variable at the head of the arrow. Symbols are defined in Table 1. Symbols in boxes are C and N stocks, symbols in circles are driver variables, and other symbols are either processes or allometric and stoichiometric constraints. The four causal links shown as dashed arrows are the links that were weakened in the calibration to accommodate vole‐mediated processes in the simulations with explicit representation of vole density (see Table 2). The temperature (*T*) and vole (*V*) drivers are shown three times to avoid overcomplicating the diagram.

Elevated CO_2_ stimulates only one ecosystem process, photosynthesis (Fig. 7). The associated increase in C gain increases biomass and leaf area, which further stimulates photosynthesis (Fig. 7: ↑*C*_a_ → ↑*P*_s_ → ↑*B*_C_ → ↑*S* → ↑*P*_s_). However, there is a much stronger negative feedback associated with the change in stoichiometry; increased photosynthesis increases biomass C and consequently increases vegetation C:N, which feeds back to decrease photosynthesis (↑*C*_a_ → ↑*P*_s_ → ↑*B*_C_ → ↑Ψ → ↓*P*_s_). Tissue and Oechell (1987) used this stoichiometric feedback to argue why tussock tundra exposed to elevated CO_2_ alone had only a transient increase in production. Without an increase in the N supply to vegetation, the CO_2_‐stimulation of photosynthesis cannot be maintained. However, the increase in vegetation C:N increases the litter‐fall C:N and consequently the soil C:N, which in turn decreases net N mineralization and the supply of N to plants (↑*C*_a_ → ↑*P*_s_ → ↑*B*_C_ → ↑Ψ → ↓*L*_itN_ → ↓*D*_N_ → ↑Φ → ↓(*N*_min_ − *U*_Nm_)). Because none of the steps in this chain were modified to incorporate voles explicitly in the model calibration, this N feedback is about the same for both explicit and aggregated representation of voles in the model and therefore does not have a large effect on the relative responses with and without explicit representation of vole effects.

### Why are the predicted responses to warming stronger when vole effects are explicitly represented in the model than when they are aggregated with other processes?

In the model, warming stimulates six processes: photosynthesis, autotrophic and heterotrophic respiration, N mineralization, microbial N uptake, and plant N uptake (Fig. 7). Although warming decreased the energy cost of thermoregulation and therefore decreases vole consumption of plants (Eq. 15) and vole respiration (Eq. 17), we found that this effect is too small to explain the differences between simulations with vs. without voles explicitly represented (accounting for <2% of the response in C and N stocks).

Among these many effects of warming in the model, the main effect that results in the accumulation of plant C in simulations with both explicit and aggregated representations of vole effects is the release from N limitation through the stimulation of net N mineralization (Fig. 7: ↑*T* → ↑(*N*_min_ − *U*_Nm_) → ↑*N* → ↑*U*_N_ → ↑*B*_N_ → ↓Ψ →↑*P*_s_). Therefore, one effect of this mobilization of soil N is for both C and N uptake by plants to increase, causing plant biomass to accumulate. Because N mineralization (*N*_min_) was decreased in calibrations II and III to accommodate the explicit representation of voles (Table 2), this warming‐induced growth in plant biomass is about 0.8% (40‐vole calibration II) to 2% (100‐vole calibration III) weaker in the simulations with the explicit representation of vole effects. These simulations nevertheless accumulate more, not less, biomass.

The main reason that plant C and N accumulation differed between simulations with aggregated vs. explicit representations of voles is the change made to litter‐fall rates to accommodate the voles in calibrations II and III (Table 2). Litter fall is not directly stimulated by warming (Eqs. 11, 12), but it does increase as plant biomass increases. This increase in litter fall in turn limits the amounts of C and N that can accumulate in plants. However, the fraction of plant C and N lost in litter fall was decreased in calibrations II and III in which voles are explicitly represented to accommodate the C and N consumed by voles (Table 2: *m*_CB_ and *m*_NB_ were decreased). Consumption by voles does not increase with plant biomass (Eq. 15), and vole density does not increase with plant biomass because we assume top‐down control on voles and therefore use a prescribed vole density. As a consequence, the increase in litter fall as biomass accumulates is smaller with voles explicitly represented than in the aggregate representation. The accumulation of C and N in vegetation is therefore larger with the explicit inclusion of vole effects than in the simulation in which vole effects are aggregated with other processes. When we do allow vole density to increase in proportion of the increase in NPP (~80% over 100 years), the increase in plant biomass is less than 8% lower because of consumption and the small increase in soil C (<0.5%) becomes a small decrease (<1%).

In addition, because the C:N ratio of forage (19.15 g C·g^−1^ N) is lower than the C:N ratio of litter (40 g C·g^−1^ N), the fraction of vegetation N lost in litter fall was decreased more than the fraction of vegetation C lost in litter fall in calibrations II and III with explicit vole representations (Table 2; e.g., 13.7% decrease in litter N vs. 6.6% decrease in litter C with 40 vole/ha). As a consequence, as vegetation biomass accumulates, the litter‐fall C:N ratio increases more with the explicit representation of voles than with aggregated representation of vole effects. Soil organic C therefore increases more with the explicit vole representation than without it (Fig. 4), and soil organic N decreases more with the explicit vole representation than without it (Fig. 5).

### Why is there a synergistic response to elevated CO_2_ and warming in combination?

If the response to CO_2_ was stronger so that there was a substantial increase in plant biomass and plant C:N ratio, then the feedback associated with litter fall would have come into play and differences between explicit and aggregated representations of vole activity would have made more of a difference by the same mechanism described above for the response to warming. When elevated CO_2_ is combined with warming, the warming mobilizes soil N, easing N limitation of plant production, and the inhibiting feedback on production associated with higher plant C:N is weakened. This weakening of the C:N feedback allows the direct effects of elevated CO_2_ to be more strongly manifested, and therefore a stronger response to both elevated CO_2_ and warming than the sum of the responses to each factor individually. Tissue and Oechell (1987) found the same synergistic effect, resulting in a sustained increase in production with elevated CO_2_ and warming but only a transient increase with CO_2_ alone.

## Conclusions

Grazing animals can have large effects on ecosystems (Grellman 2002, McLaren and Jefferies 2004, Post and Pedersen 2008, Sjögersten et al. 2008, Rinnan et al. 2009, Cahoon et al. 2011, Kaarlejärvi et al. 2015, Leffler et al. 2019, Min et al. 2021). Our simulations suggest that long‐term exclusion of voles or maintenance of vole populations at high densities can result in large gains or losses of both plants and soil C (Fig. 2). However, the response time of plants and soil to these persistent changes in grazing takes several decades in our model. As a consequence, the full effects of changes in vole densities are never realized when voles cycle on a three‐to‐four‐year time scale. Indeed, cycling at such a high frequency can be incorporated in our model using the long‐term mean density without any substantial change in the predicted long‐term dynamics of the ecosystem. Our simulations excluding and including voles are not purely academic. Recent studies suggest that the Arctic rodent population cycles could dampen in amplitude or be punctuated with periods of non‐cyclic dynamics in response to altered climate conditions, in particular changes in snow conditions (Ims and Henden 2008, Kausrud, 2008, Gilg and Sittler 2009, Brommer et al. 2010, Domine et al. 2018). Our results suggest that the important dynamics for predicting long‐term changes in tundra biogeochemistry in response to climate change are the mean grazer dynamics on decadal scales, not the higher frequency three‐to‐four‐year cycles.

The effects of voles on C and N cycling can have major effects on the biogeochemical responses of tundra to elevated CO_2_ and warming. These effects need to be explicitly represented in models rather than aggregated with other ecosystem processes. Even if these other processes act in parallel with vole processes, their response to changes in the environment can be very different. Our analysis indicates that failure to explicitly account for voles results in a large underestimation of the responses of tundra to climate warming and to elevated CO_2_ and warming. Our analysis is analogous to that of Thornton et al. (2007) who found that predicted responses of the terrestrial biosphere to elevated CO_2_ and climate change was probably overestimated unless N limitation was explicitly represented in models. We recommend that grazing effects be explicitly incorporated when applying models of tundra response to global change.

Our analysis is based on a simple, annual‐time‐step model of ecosystem C and N interactions calibrated to Arctic tundra. The simplicity of the model facilitates causal tracing (Fig. 7) and heuristic analysis of the results, but at the expense of quantitative detail in the dynamics (Rastetter 2017). The results should therefore be confirmed for more complex models with, for example, more detailed representations of vegetation and soil characteristics, finer scale seasonal dynamics, and the effects animals can have on plant‐community composition and soil structure. Although our model was calibrated for Arctic tundra, the qualitative conclusions probably apply more broadly. In our analysis, a key process is vole respiration, which decreases with warming, unlike the increase with warming for plant and microbial respiration. This property is clearly relevant to mammal grazers in cold climates, but not for mammal grazers in warm climates or for insects in any climate. Nevertheless, for these other ecosystems there might be analogous model biases associated with aggregating biogeochemical processes mediated by these grazers with other ecosystem processes. Similarly, the consequences of resource limitation need to be examined for grazers in ecosystems that are bottom‐up regulated. All these possibilities need to be analyzed, first with heuristic models such as the one we use and then incorporated into more detailed biogeochemical models. To perform these analyses, more data such as those in Batzli et al. (1980) and Olofsson et al. (2012) are needed that can be directly applied in these biogeochemical models. Collection of these data will require a biogeochemical, as well as a community, perspective on plant–grazer interactions.

## Supporting information

Appendix S1

## Data Availability

New empirical data were not used for this research. The code (Rastetter et al. 2021*a*) is available from Zenodo: http://doi.org/10.5281/zenodo.5083290 and the simulation results (Rastetter et al. 2021*b*, *c*) are available from the Environmental Data Initiative: https://doi.org/10.6073/pasta/67108cef344d93cfdd060e7e0f0911f5 and https://doi.org/10.6073/pasta/42e6660b2d1f2b59985ed0940e53f0d4

## References

[eap2478-bib-0001] Batzli, G. O. , R. G. White , S. F. MacLean Jr , F. A. Pitelka , and B. D. Collier . 1980. The herbivore‐based trophic system. Pages 335–410 *in* J. Brown , P. C. Miller , L. L. Tieszen , and F. L. Brunnell , editors. An Arctic ecosystem: the coastal tundra at Barrow, Alaska. Dowden, Hutchinson, and Ross Inc, Stroudsburg, Pennsylvania, USA.

[eap2478-bib-0002] Bennett, V. J. 2003. Computer modelling the Serengeti‐mara ecosystem. Dissertation. School of Biology, The University of Leeds.

[eap2478-bib-0003] Brommer, J. E. , H. Pietianen , K. Ahola , P. Karell , T. Karstinen , and H. Kolunen . 2010. The return of the vole cycle in southern Finland refutes generality of the loss of cycles through ‘climatic forcing’. Global Chance Biology 16:577–586.

[eap2478-bib-0004] Cahoon, S. M. P. , P. F. Sullivan , E. Post , and J. M. Welker . 2011. Large herbivores limit CO_2_ uptake and suppress carbon cycle responses to warming in West Greenland. Global Change Biology 18:469–479.

[eap2478-bib-0005] Carey, J. C. , et al. 2016. Temperature response of soil respiration largely unaltered with experimental warming. Proceedings of the National Academy of Sciences of the United States of America 113:13797–13802. 27849609 10.1073/pnas.1605365113 PMC5137763

[eap2478-bib-0006] Domine, F. , G. Gauthier , V. Vionnet , D. Fauteux , M. Dumont , and M. Barrere . 2018. Snow physical properties may be a significant determinant of lemming population dynamics in the high Arctic. Arctic Science 4:813–826.

[eap2478-bib-0007] Ehrich, D. , et al. 2020. Documenting lemming population change in the Arctic: Can we detect trends? Ambio 49:786–800. 31332767 10.1007/s13280-019-01198-7 PMC6989711

[eap2478-bib-0008] Gilg, O. , B. Sittler , and I. Hanski . 2009. Climate change and cyclic predator–prey population dynamics in the high Arctic. Global Change Biology 15:2634–2652.

[eap2478-bib-0009] Gough, L. , J. C. Moore , G. R. Shaver , R. T. Simpson , and D. R. Johnson . 2012. Above‐ and belowground responses of arctic tundra to altered soil nutrients and mammalian herbivory. Ecology 93:1683–1694. 22919914 10.1890/11-1631.1

[eap2478-bib-0010] Grellman, D. 2002. Plant responses to fertilization and exclusion of grazers on an arctic tundra heath. Oikos 98:190–204.

[eap2478-bib-0011] Hairston, N. G. , F. E. Smith , and L. B. Slobodkin . 1960. Community structure, population control, and competition. American Naturalist 94:421–425.

[eap2478-bib-0012] Heskel, M. A. , et al. 2016. Convergence in the temperature response of leaf respiration across biomes and plant functional types. Proceedings of the National Academy of Sciences of the United States of America 113:3832–3837. 27001849 10.1073/pnas.1520282113 PMC4833281

[eap2478-bib-0013] Ims, R. A. , J. A. Henden , and S. T. Killengreen . 2008. Collapsing population cycles. Trends in Ecology & Evolution 23:79–8610. 18191281 10.1016/j.tree.2007.10.010

[eap2478-bib-0014] Jai, S. , X. Wang , Z. Yuan , F. Lin , Z. Hao , and M. S. Luskin . 2018. Global signal of top‐down control of terrestrial plant communities by herbivores. Proceedings of the National Academy of Sciences of the United States of America 115:6237–6242. 29848630 10.1073/pnas.1707984115 PMC6004463

[eap2478-bib-0015] Jiang, Y. , E. B. Rastetter , G. R. Shaver , A. V. Rocha , Q. Zhuang , and B. L. Kwiatkowsk . 2017. Modeling long‐term changes in tundra carbon balance following wildfire, climate change and potential nutrient addition. Ecological Applications 27:105–117. 27898193 10.1002/eap.1413

[eap2478-bib-0016] Kaarlejärvi, E. , K. S. Hoset , and J. Olofsson . 2015. Mammalian herbivores confer resilience of arctic shrub‐dominated ecosystems to changing climate. Global Change Biology 21:3379–3388. 25967156 10.1111/gcb.12970

[eap2478-bib-0017] Kausrud, K. L. , et al. 2008. Linking climate change to lemming cycles. Nature 456:93–97. 18987742 10.1038/nature07442

[eap2478-bib-0018] Korpimaki, E. , P. R. Brown , J. Jacobs , and R. P. Pech . 2004. The puzzles of population cycles and outbreaks of small mammals solved? BioScience 54:1071–1079.

[eap2478-bib-0019] Krebs, C. J. 2013. Population fluctuations in rodents. University of Chicago Press, Chicago, Illinois, USA.

[eap2478-bib-0020] Krebs, C. J. , R. Boonstra , and A. J. Kenney . 1995. Population dynamics of the collared lemming and the tundra vole at Pearce Point, Northwest Territories, Canada. Oecologia 103:481–489. 28306997 10.1007/BF00328687

[eap2478-bib-0021] Leffler, A. J. , K. H. Beard , K. C. Kelsey , R. T. Choi , J. A. Schmutz , and J. M. Welker . 2019. Delayed herbivory by migratory geese increases summer‐long CO_2_ uptake in coastal western Alaska. Global Change Biology 25:277–289. 30295398 10.1111/gcb.14473

[eap2478-bib-0022] Legagneux, P. , et al. 2012. Disentangling trophic relationships in a high arctic tundra ecosystem through food web modeling. Ecology 93:1707–1716. 22919916 10.1890/11-1973.1

[eap2478-bib-0023] McGuire, A. D. , et al. 2012. An assessment of the carbon balance of arctic tundra: Comparisons among observations, process models, and atmospheric inversions. Biogeosciences 9:3185–3204.

[eap2478-bib-0024] McKane, R. , E. Rastetter , G. Shaver , K. Nadelhoffer , A. Giblin , J. Laundre , and F. Chapin . 1997. Reconstruction and analysis of historical changes in carbon storage in arctic tundra. Ecology 78:1188–1198.

[eap2478-bib-0025] McKinnon, L. , P. A. Smith , E. Nol , J. L. Martin , F. I. Doyle , K. F. Abraham , H. G. Gilchrist , R. I. G. Morrison , and J. Bêty . 2010. Lower predation risk for migratory birds at high latitudes. Science 327:326–327. 20075251 10.1126/science.1183010

[eap2478-bib-0026] McLaren, J. R. , and R. L. Jefferies . 2004. Initiation and maintenance of vegetation mosaics in an Arctic salt marsh. Journal of Ecology 92:648–660.

[eap2478-bib-0027] McNaughton, S. J. 1985. Ecology of a grazing ecosystem: The Serengeti. Ecological Monographs 55:259–294.

[eap2478-bib-0028] Metcalfe, D. B. , et al. 2014. Herbivory makes major contributions to ecosystem carbon and nutrient cycling in tropical forests. Ecology Letters 17:324–332. 24372865 10.1111/ele.12233

[eap2478-bib-0029] Metclafe, D. B. , and J. Olofsson . 2015. Distinct impacts of different mammalian herbivore assemblages on arctic tundra CO_2_ exchange during the peak of the growing season. Oikos 124:1632–1638.

[eap2478-bib-0030] Min, E. , M. Wilcots , S. Naeem , L. Gough , J. R. McLaren , R. J. Rowe , E. Rastetter , N. Boelman , and K. L. Griffin . 2021. Herbivore absence can shift dry heath tundra from carbon source to sink during peak growing season. Environmental Research Letters 16:024027.

[eap2478-bib-0031] Oli, M. 2019. Population cycles in voles and lemmings: state of the science and future directions. Mammal Review 49:226–239.

[eap2478-bib-0032] Olofsson, J. , H. Tømmervik , and T. V. Callaghan . 2012. Vole and lemming activity observed from space. Nature Climate Change Letters 2:880–883.

[eap2478-bib-0033] Pastor, J. R. , J. Naiman , B. Dewey , and P. McInnes . 1988. Moose, microbes and the boreal forest. BioScience 38:770–779.

[eap2478-bib-0034] Pearce, A. R. , E. B. Rastetter , W. B. Bowden , M. C. Mack , Y. Jiang , and B. L. Kwiatkowski . 2015. Recovery of arctic tundra from thermal erosion disturbance is constrained by nutrient accumulation: a modeling analysis. Ecological Applications 25:1271–1289. 26485955 10.1890/14-1323.1

[eap2478-bib-0035] Petit Bon, M. , K. G. Inga , I. S. Jónsdóttir , T. A. Utsi , E. M. Soininen , and K. A. Bråthen . 2020. Interactions between winter and summer herbivory affect spatial and temporal plant nutrient dynamics in tundra grassland communities. Oikos 129:1229–1242.

[eap2478-bib-0036] Pitelka, F. A. , and G. O. Batzli . 2007. Population cycles of lemmings near Barrow, Alaska: a historical review. Acta Theriologica 52:323–336.

[eap2478-bib-0037] Pitelka, F. A. , P. Q. Tomich , and G. W. Treichel . 1955. Ecological relations of jaegers and owls as lemming predators near Barrow, Alaska. Ecological Monographs 25:85–117.

[eap2478-bib-0038] Post, E. , and C. Pedersen . 2008. Opposing plant community responses to warming with and without herbivores. Proceedings of the National Academy of Sciences of the United States of America 105:12353–12358. 18719116 10.1073/pnas.0802421105 PMC2527915

[eap2478-bib-0039] Prevedello, J. M. , C. R. Dickman , M. V. Vieira , and E. M. Vieira . 2013. Population responses of small mammals to food supply and predators: a global meta‐analysis. Journal of Animal Ecology 82:927–936. 23560951 10.1111/1365-2656.12072

[eap2478-bib-0040] Rastetter, E. B. 2017. Modeling for understanding v. modeling for numbers. Ecosystems 20:215–221.

[eap2478-bib-0041] Rastetter, E. B. , G. I. Ågren , and G. R. Shaver . 1997. Responses of N‐limited ecosystems to increased CO_2_: A balanced‐nutrition, coupled‐element‐cycles model. Ecological Applications 7:444–460.

[eap2478-bib-0042] Rastetter, E. , K. Griffin , R. Rowe , L. Gough , J. McLaren , and N. Boelman . 2021a. ARC‐LTER/vole: Initial release ‐ VOLE v4.0 (Version v4.0). Zenodo. 10.5281/zenodo.5083290

[eap2478-bib-0043] Rastetter, E. , K. Griffin , R. Rowe , L. Gough , J. McLaren , and N. Boelman . 2021b. Modeling the effect of explicit vs implicit representation of grazing on ecosystem carbon and nitrogen cycling in response to elevated carbon dioxide and warming in arctic tussock tundra, Alaska ‐ Dataset A ver 2. Environmental Data Initiative. 10.6073/pasta/67108cef344d93cfdd060e7e0f0911f5

[eap2478-bib-0044] Rastetter, E. , K. Griffin , R. Rowe , L. Gough , J. McLaren , and N. Boelman . 2021c. Modeling the effect of explicit vs implicit representation of grazing on ecosystem carbon and nitrogen cycling in response to elevated carbon dioxide and warming in arctic tussock tundra, Alaska ‐ Dataset B ver 2. Environmental Data Initiative. 10.6073/pasta/42e6660b2d1f2b59985ed0940e53f0d4

[eap2478-bib-0045] Rastetter, E. B. , G. W. Kling , G. R. Shaver , B. C. Crump , L. Gough , and K. L. Griffin . 2020. Ecosystem recovery from disturbance is constrained by N cycle openness, vegetation‐soil N distribution, form of N losses, and the balance between vegetation and soil‐microbial processes. Ecosystems 24:667–685.

[eap2478-bib-0046] Rinnan, R. , S. Stark , and A. Tolvanen . 2009. Responses of vegetation and soil microbial communities to warming and simulated herbivory in a subarctic heath. Journal of Ecology 97:788–800.

[eap2478-bib-0048] Schmitz, O. J. , et al. 2014. Animating the carbon cycle. Ecosystems 17:344–359.

[eap2478-bib-0049] Seagle, S. W. , and S. J. McNaughton . 1993. Simulated effects of precipitation and nitrogen on Serengeti grassland productivity. Biogeochemistry 22:157–178.

[eap2478-bib-0050] Sjögersten, S. , R. van der Wal , and S. J. Woodin . 2008. Habitat type determines herbivory controls over CO_2_ fluxes in a warmer Arctic. Ecology 89:2103–2116. 18724721 10.1890/07-1601.1

[eap2478-bib-0051] Thornton, P. E. , J. F. Lamarque , N. A. Rosenbloom , and N. M. Mahowald . 2007. Influence of carbon‐nitrogen cycle coupling on land model response to CO_2_ fertilization and climate variability. Global Biogeochemical Cycles 21:GB4018.

[eap2478-bib-0052] Tissue, D. T. , and W. C. Oechell . 1987. Response of *Eriophorum* *vaginatum* to elevated CO_2_ and temperature in the Alaskan tussock tundra. Ecology 68:401–410.

[eap2478-bib-0053] Tuomi, M. , S. Stark , K. S. Hoset , M. Väisänen , L. Oksanen , F. J. A. Murguzur , H. Tuomisto , J. Dahlgren , and K. A. Bråthen . 2019. Herbivore effects on ecosystem process rates in a low‐productive system. Ecosystems 22:827–843.

[eap2478-bib-0054] Wardle, D. A. , R. D. Bardgett , J. N. Klironomos , H. Setälä , W. H. van der Putten , and D. H. Wall . 2004. Ecological linkages between aboveground and belowground biota. Science 304:1629–1633. 15192218 10.1126/science.1094875

[eap2478-bib-0055] Wilkinson, D. M. , and T. N. Sherratt . 2016. Why is the world green? The interactions of top–down and bottom–up processes in terrestrial vegetation ecology. Plant Ecology and Diversity 9:127–140.

[eap2478-bib-0056] Yu, Q. , H. E. Epstein , D. A. Walker , G. V. Frost , and B. C. Forbes . 2011. Modeling dynamics of tundra plant communities on the Yamal Peninsula, Russia, in response to climate change and grazing pressure. Environmental Research Letters 6:045505.

[eap2478-bib-0057] Zimov, N. S. , S. A. Zimov , A. E. Zimova , G. H. Zimova , V. I. Chuprynin , and F. S. Chapin III . 2009. Carbon storage in permafrost and soils of the mammoth tundra‐steppe biome: Role in the global carbon budget. Geophysical Research Letters 36:L02502.

