# Supplementary material for: Model responses to CO2 and warming are underestimated without explicit representation of Arctic small‐mammal grazing
Source: Ecol Appl. 2021 Dec 8;32(1):e02478. doi: 10.1002/eap.2478 (PMC9285540; doi:10.1002/eap.2478)
Supplement: Supplementary file 1 — Appendix S1 [file EAP-32-0-s001.pdf]

Supporting Information. Rastetter, E. B., K. L. Griffin, R. J. Rowe, L. Gough, J. R. McLaren, and N. T. Boelman. 2021. Model responses to CO_2 and warming are underestimated without explicit representation of Arctic small-mammal grazing. Ecological Applications.

Appendix S1: Derivation of stocks, fluxes and parameter values.

Section S1: Calibration for the aggregated model (vole activity not explicitly represented)

We set C and N stocks and fluxes to be consistent with data collated by Pearce et al. (2015) for the Multiple Element Limitation (MEL) model applied to tussock tundra (Table 1 in main text). Our detritus C and N are the aggregated value of the three detritus stocks the MEL model. To compensate for differences in model structures, we calculated litter-fall C and N (L_{itC} and L_{itN}), heterotrophic respiration (R_h), N immobilization (U_{Nm}), and N inputs (N_{in}) by difference assuming the ecosystem was in steady state.

We derived parameter values from various sources. We fit our allometric parameters (α and γ) to the MEL allometric equation for tundra from Pearce et al. (2015). We assume a stoichiometric balance for calibration and therefore set the optimum vegetation and detritus C:N ratios (q_B and q_D) to the C:N ratios of the respective stocks from Pearce et al. (2015). Similarly, we set the C:N of dissolved organic matter losses (q_{DOM}) to the ratio of the respective fluxes estimated from Pearce et al. (2015). To mimic the CO_2 response reported in Tissue and Oechell (1987), we set the CO_2 half-saturation constant for photosynthesis (k_C) to 100 μmol mol^{-1}. To impose strong N limitation (Shaver et al. 2014), we set both the half saturation constants for plant (k_N) and microbial (k_{Nm}) N uptake to 1 g N m^{-2} as in Rastetter et al. (2020).

We also set the temperature responses based on data from various sources. For photosynthesis we use a Q_{10} value of 1.55, which is a median of values derived from data in Tieszen (1973) and Rogers et al. (2019). For autotrophic respiration we use a Q_{10} of 2.7 based on a fit between 10 and 15 °C to the model for tundra species in Heskel et al. (2016). For heterotrophic respiration we use a Q_{10} of 3 again based on a fit between 10 and 15 °C to the model for Boreal forest soils in Carey et al. (2016). Atkin and Cummins (1994) report ^{15}N-based uptake rates for arctic plants consistent with Q_{10} values ranging from 1.16 to 3.17. Dong et al. (2001) and Yan et al. (2012) report plant N uptake rates in an agricultural setting consistent with Q_{10} values of 3.99 and 1.67. Based on these studies, we assume a Q_{10} of 2 for N uptake by plants. For microbial N uptake (immobilization), we use a Q_{10} value of 1.95 as reported by Roberts and Jones (2012) for microbial uptake of amino sugars. Finally, for N mineralization, Roberts and Jones (2012) report a Q_{10} value of 2.32 and Vinolas et al. (2001) report a value of 2; we use a Q_{10} value of 2.16.

Except for parameters associated with vole activity, the only parameters remaining are the rate parameters for each of the C and N fluxes. We calibrate these rate parameters to the process rates reported in Pearce et al. (2015) for the MEL model applied to tussock tundra (Table 1 in main text).

Section S2:Calibration of the distributed model (explicit representation of vole activity)

We assume generic small mammals (voles and lemmings) weighing 50 g, but will refer to them as a "voles." We estimate C removal from vegetation by voles from two processes, consumption and nest building. The consumption rates depend on an allometric relation to body weight minus a correction for temperature to compensate for the energy needed to maintain body

temperature (Batzli et al. 1980). We modify this function to account for the difference between the summer temperature we use to drive the model and subnivean temperature during the winter. We assume that the average annual temperature experienced by the voles is ten degrees cooler than the average summer temperature:

$$E = 26.82 + 5.36\, W^{0.75} - 1.89\,(T - 10)$$

where E is daily energy expenditure (kJ/vole/day), W is body weight (g fresh weight/vole), T is mean summer temperature (ºC). For a 50 g vole, this equation simplifies to

$$E = 127.6 - 1.89\,(T - 10)$$

Total food assimilation must meet this energy expenditure, but only about 33% of ingested forage gets assimilated (Batzli et al. 1980). Thus, total ingestion must contain about three times this amount of energy. To convert this ingestion to g C vole^{-1} yr^{-1}, we assume a forage energy density of 18.9 kJ/g dry weight (Batzli et al. 1980) and a C density of forage of 0.475 g C/g dry weight (Schlesinger 1991):

$$I = 3512 - 52\,(T - 10)$$

where I is ingestion (g C vole^{-1} yr^{-1}). Thus, for the ingestion part of Eq. 15 (Box 1 in main text)

$$g_V = 3512 \text{ g C vole}^{-1}\text{ yr}^{-1}, \quad \varepsilon_V = 52 \text{ g C ºC}^{-1}\text{ vole}^{-1}\text{ yr}^{-1}, \text{ and } \quad T_0 = 10 \text{ ºC}$$

Voles and lemmings also remove plant material to build winter nests. Vole nests contain about 20 g C nest^{-1} (Rowe unpub. data). Krebs et al. (2012) estimate approximately 2.2 nests per lemming in the spring. Data from Maguire and Rowe (2017) indicate that singing vole density in spring is about half the average annual density. We therefore estimate that in addition to ingestion, our generic small mammal (vole) grazer removes 22 g C vole^{-1} yr^{-1} from the vegetation for nests. We add this nest C to the ingestion equation to get the final parameter for Eq. 15:

$$n_V = 22 \text{ g C vole}^{-1} \text{ yr}^{-1}$$

Respiration is about 30% of ingestion (Batzli et al. 1980):

$$r_V = 0.3 \text{ g C g}^{-1} \text{ C}$$

Forage contains about 25 mg N/g dry weight (Batzli et al. 1980), which is equivalent to a C:N ratio of 19 g C g^{-1} N. We assume the C:N ratio of the nest material is the same as that of the vegetation (42.62 g C/g N). We calculate the C:N of material removed from vegetation by voles as the weighted mean of these two C:N ratios:

$$q_V = \frac{3512 \times 19 + 22 \times 42.62}{3534} = 19.15 \text{ g C g}^{-1} \text{ N}$$

We set the per capita urine N production based on data for small mammals reported by Clark et al. (2005):

$m_{NV} = 11$ g N vole^{-1} yr^{-1}

Section S3: LITERATURE CITED

Atkin, O. K., and W. R. Cummins. 1994. The effect of root temperature on the induction of nitrate reductase activities and nitrogen uptake rates in arctic plant species. Plant ans Soil 159:187-197.

Batzli, G. O., R. G. White, S. F. MacLean Jr, F. A. Pitelka, and B. D. Collier. 1980. The herbivore-based trophic system. pp 335-410 in Brown, J., Miller, P.C., Tieszen, L.L., and Brunnell, F.L. eds.. An Arctic Ecosystem: the Coastal Tundra at Barrow, Alaska. Dowden, Hutchinson, and Ross Inc, Stroudsburg, PA, USA

Carey, J. C., J. Tang, P. H. Templar, K. D. Kroger, T. W. Crowther, A. J. Burton, J. S. Dukes, B. Emmett, S. D. Frey, M. A. Heskel, L. Jiang, M. B. Machmuller, J. Mohan, A. M. Panetta, P. B. Reich, S. Reinsch, X. Wang, S. D. Allison, C. Bamminger, S. Bridgham, S. L. Collins, G. de Dato, W. C. Eddy, B. J. Enquist, M. Estiarte, J. Harte, A. Henderson, B. R. Johnson, K. S. Larsen, Y. Luo, S. Marhan, J. M. Melillo, J. Peñuelas, L. Pfeifer-Meister, C. Poll, E. Rastetter, A. B. Reinmann, L. L. Reynolds, I. K. Schmidt, G. R. Shaver, A. L. Strong, V. Suseela, and A. Tietema. 2016. Temperature response of soil respiration largely unaltered with experimental warming. Proc Nat Accad Sci 113:13797-13802.

Clark, J. E., E. C. Hellgren, J. L. Parsons, E. E. Jorgensen, D. M. Engle, and D. M. Lesli Jr. 2005. Nitrogen outputs from fecal and urine deposition of small mammals: implications for nitrogen cycling. Oecologia 144:447-455.

Dong, S., C. F. Scagel, L. Cheng, L. H. Fuchigami, and P. T. Rygiewicz. 2001. Soil temperature and plant growth stage influence nitrogen uptake and amino acid concentration of apple during early spring growth. Tree Phys. 21:541-547.

Heskel, M. A., O. S. O'Sullivan, P. B. Reich, M. G. Tjoelker, L. K. Weerasinghe, A. Penillard, J. J. G. Egerton, D. Creek, K. J. Bloomfield, J. Xiang, F. Sinca, Z. R. Stangl, A. Martinez-de la Torre, K. L. Greffin, C. Huntingford, V. Hurry, P. Meir, M. H. Turnbull, and O. K. Atkin. 2016. Convergence in the temperature response of leaf respiration across biomes and plant functional types. Proc Nat Accad Sci 113: 3832-3837.

Krebs, C. J., F. Bilodeau, D. Reid, G. Gauthier, A. J. Kenney, S. Gilbert, D. Duchesne, and D. J. Wilson. 2012. Are lemming winter nest counts a good index of population density? J Mammalogy 93:87-92.

Maguire, A. J., and R. J. Rowe. 2017. Home range and habitat affinity of the singing vole on the North Slope of Alaska. Arctic, Antarctic, and Alpine Research 492.:243–257.

Pearce, A. R., E. B. Rastetter, W. B. Bowden, M. C. Mack, Y. Jiang, Y., and B. L. Kwiatkowski, B.L. 2015. Recovery of arctic tundra from thermal erosion disturbance is constrained by nutrient accumulation: a modeling analysis. Ecological Applications 25:1271-1289.

Rastetter, E. B., G. W. Kling, G. R. Shaver, B. C. Crump, L. Gough, and K. L. Griffin. 2020. Ecosystem recovery from disturbance is constrained by N cycle openness, vegetation-soil N distribution, form of N losses, and the balance between vegetation and soil-microbial processes. Ecosystems https://doi.org/10.1007/s10021-020-00542-3

Roberts. P., and D. L. Jones. 2012. Microbial and plant uptake of free amino sugars in grassland soil. Soil Biology Biochemistry 49:139-149.

Rogers, A., S. P. Serbin, K. S. Ely, and S. D. Wullschleger. 2019. Terrestrial biosphere models may overestimate arctic CO_2 assimilation if they do not account for decreased quantum yield and convexity at low temperature. New Phytologist 223:167-179.

Schlesinger, W. H. 1991. Biogeochemistry: An Analysis of Global Change. Academic Press, San Diego, CA, USA.

Shaver, G. R., J. A. Laundre, M. S. Bret-Harte, F. S. Chapin, III, J. A. Mercado- Diaz, A. E. Giblin, L. Gough, W. A. Gould, S. E. Hobbie, G. W. Kling, M. C. Mack, J. C. Moore, K. J. Nadelhoffer, E. B. Rastetter, and J. P. Schimel, J.P. 2014. Terrestrial Ecosystems at Toolik Lake, Alaska. In Hobbie, J.E., and G. W. Kling (eds.). A Changing Arctic: Ecological Consequences for Tundra, Streams and Lakes. Oxford University Press, New York, NY, USA.

Tieszen, L. L. 1973. Photosynthesis and respiration in arctic tundra grasses: field light intensity and temperature responses. Arctic Alpine Res 5:239-251.

Tissue, D. T., and W. C. Oechell. 1987. Response of *Eriophorum vaginatum* to elevated CO2 and temperature in the Alaskan tussock tundra. Ecology 68:401-410.

Vinolas, L. C., V. R. Vallejo, and D. L. Jones. 2001. Control of aminoacid mineralization and microbial metabolism by temperature. Soil Biology Biochemistry 33:1137-1140.

Yan, Q., Z. Duan, J. Mao, X. Li, and F. Dong. 2012. Effects of root-zone temperature and N, P, and K supplies on nutrient uptake of cucumber *Cucumis sativus L.*. seedlings in hydroponics. Soil Science and Pland Nutrition 58:707-717.
